# Measurement of population agglomeration, dynamic change characteristics, and motivations in metropolitan agglomerations—A case study of the Xi’an metropolitan area

**DOI:** 10.1371/journal.pone.0316385

**Published:** 2025-01-29

**Authors:** Ke Liu, Xu Bo, Wang Zhaoping, Ran Du, Chen Heng

**Affiliations:** 1 International Business School, Shaanxi Normal University, Xi’an, Shaanxi, China; 2 School of Economics, Huazhong University of Science and Technology, Wuhan, China; 3 School of Management, Xi’an Polytechnic University, Xi’an, Shaanxi, China; National University of Sciences and Technology, PAKISTAN

## Abstract

This article compares the population agglomeration characteristics of the Xi’an metropolitan area in western China with those of metropolitan areas in other regions officially approved by the Chinese government. The kernel density estimation method and Markov chain model were used to conduct the study. The results revealed that from 2010 to 2020, the population agglomeration level of the Xi’an metropolitan area showed a trend of first increasing and then decreasing. The absolute gap in the population agglomeration level between cities within the metropolitan area gradually narrowed, and the polarization phenomenon of population agglomeration was not obvious. Compared with metropolitan agglomerations such as Nanjing, Wuhan, Fuzhou, Changsha-Zhuzhou-Xiangtan, Chongqing, and Chengdu, the Xi’an metropolitan agglomeration had a lower population agglomeration level, with a significant gap. Moreover, there was an obvious “club convergence” phenomenon in the population agglomeration levels of different urban agglomerations. The probability of the population agglomeration level remaining stable was at least 53.85%, indicating that there was a “Matthew effect” in which the rich become richer and the poor become poorer. Through the convergence models of α and β, the analysis suggested that there was no significant α convergence between the population agglomeration level of the Xi’an metropolitan agglomeration and that of other metropolitan agglomerations. Instead, there was a significant β divergence, indicating that the gap between the Xi’an metropolitan agglomeration’s population agglomeration level and that of other metropolitan agglomerations is gradually widening. An integrated theoretical framework of population agglomeration was constructed from three dimensions: producers, consumers, and social people. An empirical analysis was conducted on the causes of population agglomeration in the Xi’an metropolitan area and other metropolitan areas. The multiple regression results showed that the income level, public consumption expenditure level, education level, comfortable living environment, and educational level were important factors leading to differences in population agglomeration. The geographic detector results showed that factors in the consumer dimension were the main reasons for population agglomeration in metropolitan areas.

## 1. Introduction

After the 1980s, some developed countries began to experience negative population growth [1]. Since the beginning of the 21st century, global population development has undergone major changes, and the populations of Russia, Poland, Japan and other countries have gradually shown a negative growth trend. On November 15, 2022, the United Nations announced that the world population had reached 8 billion. This is an important milestone in the history of population development. Dr. Tomas Sobotka, an expert at the Vienna Institute of Demography, believes, “It symbolizes the end of the era of rapid population growth. More and more countries are experiencing zero or negative population growth. Compared to the past, we are facing new challenges.” As a major world population, China witnessed a population decline in 2022, officially entering an era of negative population growth. Given this situation, the impact of population issues on regional development has attracted much attention.

With the rapid development of the market economy, the urbanization process is accelerating, and many people are moving between different cities. According to data from the seventh national census, the total number of migrants in China reached 376 million in 2020, accounting for 26.6% of the total population. The large scale of the migrant population has a far-reaching impact on the development patterns of Chinese cities. With the advent of the era of negative population growth, China’s urban structure will be restructured, and the population concentration in large cities will become more obvious. The population factor is the core driving force that determines the future development of urbanization. With the proposal of China’s “14th Five-Year Plan”, the construction of modern metropolitan areas will be the focus of the new development stage. The “Guiding Opinions on Cultivating and Developing Modern Metropolitan Areas” noted that metropolitan areas are urbanized spatial forms centered on superlarge and extralarge cities with a 1-hour commuting circle as the basic scope. In recent years, research on metropolitan areas has increased significantly. Some scholars have researched the Tokyo metropolitan area, the Yangtze River Delta region, and the Guangdong–Hong Kong–Macao Greater Bay Area [2–4]. On March 25, 2022, the Shaanxi Provincial People’s Government officially issued the “Xi’an Metropolitan Area Development Plan”, pointing out that it is necessary to optimize the spatial pattern of the development of the Xi’an Metropolitan Area, to accelerate industrial and population agglomeration, and to increase efforts to attract talent. The Xi’an Metropolitan Area is located in the core area of the Silk Road Economic Belt of the “Belt and Road Initiative”, is a strategic fulcrum for the construction of the “Belt and Road Initiative”, and is also a key area in China’s “two horizontal and three vertical” urbanization strategic pattern.

The population is a key factor in urban economic development. Studying population agglomeration is highly important for promoting population development and achieving social progress. Therefore, against the background of negative population growth, this study systematically constructs a population agglomeration research framework of population agglomeration level indicators, dynamic evolution characteristics, and motivation analysis to analyze the dynamic evolution trend and motivation of population agglomeration in the Xi’an Metropolitan Area, China. This study has important strategic and practical significance for improving the quality of population agglomeration in the Xi’an Metropolitan Area, revitalizing human resources, improving labor productivity, maintaining the vitality and sustainable and healthy development of the metropolitan area, promoting high-quality economic development in the metropolitan area, and improving the level of urbanization. Moreover, it provides a complete framework for developing countries to study the problem of population agglomeration in metropolitan areas and provides case evidence for promoting the sustainable development of populations and cities.

## 2. Literature review

### 2.1. Concept and measurement of population agglomeration

With the rapid development of the market economy, the urbanization process continues to accelerate, and many people move between different cities. Population concentration is not only the static result of population movement but also a dynamic process in which labor resources are optimized in market allocation [2]. Population concentration refers to the phenomenon where a certain geographical area attracts some or even all of the population based on various factors [5,6]. Population concentration occurs when certain specific regions within a country have a relatively high proportion of the total population, creating a relative advantage in terms of the population size [7]. In terms of research methods, many scholars use single indicators such as the population density, number of permanent residents, the population-to-land ratio, and population geographic concentration to measure population concentration [8–13]. Some scholars use composite indicators to measure population concentration. For instance, Krugman (1991) proposed a “labor market aggregation model” in which firms adjust their production and employment levels in response to idiosyncratic productivity shocks [14]. The model suggests that firms tend to be spatially concentrated in locations where there are many workers with the skills required for production. Cheng K. and Hong Z. used nighttime light data to identify metropolitan areas closely related to population and land, constructing a “metropolitan population aggregation index” to measure population concentration [15]. Liu G. and Han S. selected five secondary indicators, including population income structure, population education structure, urban–rural population structure, population employment structure, and population age structure, to characterize population concentration [16].

### 2.2. Research on factors affecting population agglomeration

Ravenstein categorized factors influencing population concentration into two main categories: exogenous factors, such as wars and disasters, and endogenous factors, including differences in income and employment opportunities [17]. Lee, on the basis of “push-pull” theory, explained population migration behavior and suggested that disparities in income levels are among the driving factors for population movement [18]. Duncan and Newman divided the motivations for population concentration into consumptive and productive migration motivations. Productive migration pertains to income and economic opportunities, whereas consumptive migration aims to enhance the quality of residence or the community environment. In the United States, the motivation for the majority of immigrants is consumptive in nature [19]. Scholars have further utilized the concept of “quality of life” to explain the motivations behind population migration. For example, Hsieh and Liu argued that differences in quality of life and life satisfaction between regions are important factors leading to population migration [20]. Factors such as social security, educational resources, environmental quality, resident qualifications, urban infrastructure, cultural differences, and social status have gradually become catalysts for population concentration. Arntz reported that factors driving spatial mobility include both monetary and nonmonetary factors, such as natural comfort, consumption comfort, and public comfort [21]. Stark introduced “relative deprivation theory” to explain the motivations of migrants, suggesting that relative deprivation is one of the influencing factors for migration [22]. Migration, as a selection mechanism, occurs because capable individuals have a stronger motivation for upward social mobility [23]. Christina et al. analyzed the relationship between job security and immigration. Cai et al. suggested that the key to achieving labor transfer requires the gradual removal of institutional barriers [24]. Urban economists, based on “voting with their feet” theory, have found that regions with a high quality of life are more likely to attract population concentration [25]. Natural comfort, a component of quality of life, promotes population agglomeration [26].

### 2.3. Research on metropolitan areas

The agglomeration of the population into metropolitan cities has become a trend in population development. In China, regional population concentration is characterized by supply-side human capital, advanced labor division, and demand-side population aggregation, overlaying innovative development to drive expanded and upgraded consumption demand [6,27]. With the introduction of the “14th Five-Year Plan” in China, the construction of modern metropolitan areas has been highlighted as a key focus for the new development stage. In recent years, Chinese scholars have made significant advancements in the study of population concentration in metropolitan area. Scholars have conducted research on topics such as the modern metropolitan areas in China, the Tokyo metropolitan area, the Yangtze River Delta region, the Guangdong–Hong Kong–Macao Greater Bay Area, etc. [2–4].

In recent years, with the development of nighttime light data, some scholars have applied light data to conduct scientific research in different fields, such as inverting economic growth, population spatialization, carbon emissions, and urban built-up areas [28–30]. Some scholars have also introduced light data into urban agglomerations to study their spatial structure, analyzed the spatiotemporal dynamic evolution characteristics of urban agglomerations, and conducted research on cities by describing urban entities on the basis of light data [31–33].

### 2.4. Literature comment

Current research on population agglomeration is characterized by stronger theoretical foundations and broader perspectives. It primarily investigates the distribution characteristics of population agglomeration from a macroscopic viewpoint. Traditional indicators such as the population density are chosen to measure population agglomeration, and lighting data are rarely used to measure urban population factors. However, there is a scarcity of research results related specifically to population agglomeration in developing countries, and there is a lack of measurement and dynamic recognition of the population agglomeration in the Xi’an metropolitan area, a historical city in China. The perspective of social mobility research has not received adequate attention. With rapid socioeconomic changes taking place, populations are actively participating in a mobile society. Therefore, the motivations for population agglomeration not only include economic, consumption and social factors but also a comprehensive reflection of multiple factors. However, existing research has not integrated these three aspects into a comprehensive social mobility framework, nor has it analyzed their integrated impact on population migration and decision-making motivations.

Therefore, the main contributions of this study are as follows. First, we selected samples from various regions in the Xi’an metropolitan area of China, used nighttime light data and population density data to screen real urban areas, and further measured the population concentration in real areas, providing quantitative evidence for population agglomeration in the metropolitan areas of other countries.

Second, using kernel density estimation and the Markov chain model, we compared the dynamic evolution characteristics and trends of population agglomeration in the Xi’an metropolitan area with other metropolitan areas officially approved by the National Development and Reform Commission of China, providing ideas and models for analyzing the dynamic evolution characteristics of population agglomeration in metropolitan areas of other countries.

Third, on the basis of social mobility, socioeconomic stratification theory and Maslow’s hierarchy of needs theory, an integrated theoretical framework for population agglomeration motivation is constructed, and the motivations for the population agglomeration differences between the Xi’an Metropolitan Area and other metropolitan areas are analyzed from the three dimensions: producers, consumers and social people. This provides a theoretical framework and ideas for developing countries to analyze the motivations for population agglomeration in metropolitan areas.

## 3. Models and methods

### 3.1. Sample selection and data sources

This study selected the Xi’an metropolitan area in China as the research sample. According to official documents, the Xi’an metropolitan area centered on Xi’an mainly includes Xi’an’s Xincheng District, Beilin District, Lianhu District, Baqiao District, Weiyang District, Yanta District, and Yan District. Liang District, Lintong District, Chang’an District, Gaoling District, Huyi District, Lantian County and Zhouzhi County. Yaozhou District of Tongchuan City, Qindu District, Yangling District, Weicheng District, Jingyang County, Sanyuan County, Qian County, Liquan County, Wugong County and Xingping City of Xianyang City, Linwei District, Huazhou District and Fuping County of Weinan City.

The Nanjing metropolitan area mainly includes the entire areas of Nanjing, Zhenjiang, Yangzhou, Huai’an, Wuhu, Ma’anshan, Chuzhou and Xuancheng, as well as Jintan District and Liyang City in Changzhou. The Fuzhou metropolitan area mainly includes Fuzhou, Putian, Yanping District of Nanping, Jianyang District of Nanping, Jian’ou City of Nanping, Jiaocheng District of Ningde, Xiapu County, Gutian County and Fu’an City. The Wuhan metropolitan area mainly includes Wuhan, Ezhou, Huangshi and Huanggang. The Changzhutantan metropolitan area covers the entire area of Changsha, the central urban areas of Zhuzhou and Liling, and the central urban areas of Xiangtan, Shaoshan City and Xiangtan County. The Chongqing Metropolitan Area includes 21 districts in Chongqing, including Yuzhong District, Dadukou District, Jiangbei District, Shapingba District, Jiulongpo District, Nan’an District, Beibei District, Yubei District, Banan District, Fuling District, Changshou District, Jiangjin District, Hechuan District, Yongchuan District, Nanchuan District, Qijiang District-Wansheng Economic Development Zone, Dazu District, Bishan District, Tongliang District, Tongnan District, Rongchang District, and Guang’an City in Sichuan Province. The Chengdu Metropolitan Area is centered on Chengdu City, and is composed of Deyang City, Meishan City, and Ziyang City, which are closely connected.

In this study, various regions within the Xi’an metropolitan area in China were selected as the research samples. The population agglomeration data were extracted from the WorldPop population density data for the years 2010 to 2022 and converted into panel data via the R programming language. The light data were obtained from SNPP-VIIRS and DMSP-OLS sources. The SNPP-VIIRS data were transformed into simulated DMSP‒OLS data via the R programming language. The calibrated DMSP‒OLS data from 2010 to 2013 were merged with the simulated DMSP‒OLS data from 2013 to 2022 to create a dataset resembling the DMSP‒OLS data for the period from 2010 to 2022. Other economic data primarily came from sources such as the China Statistical Yearbook, the Population Statistical Yearbook, the Urban Statistical Yearbook, the Guo Taian database, the Wind database, etc. Interpolation methods were used to fill in missing data. Data processing was conducted via software such as STATA 17.0 and MATLAB. To ensure data consistency, this study utilized data up to 2022, as some county-level yearbook data were available only up to that year.

### 3.2. Methods

#### 3.2.1. Population agglomeration measurement method.

Given that population agglomeration in different metropolitan areas comprises attributes related to both population size and land area, this study draws on the research of Cheng and Hong [15]. It constructs a population concentration index using data from both nighttime light and population distributions to measure population agglomeration. To maintain consistency with the sample period of economic panel data from 2010 to 2022, two sources of nighttime light data are used, including DMSP-OLS and SNPP-VIIRS nighttime light data. The demographic data used global population raster data from 2010 to 2022 provided by the WorldPop database.

The calculation of the population agglomeration index involves selecting areas that meet the criteria of “nighttime light intensity exceeding a certain threshold” and “population density exceeding a certain level” to be considered actual population agglomeration areas. When determining the realization area from 2010 to 2022, we draw lessons from the practices of Cheng and Hong [15], and Qin et al. [34]. This method selects areas that simultaneously meet the criteria of population density greater than 1000 people/km^2^ and nighttime light intensity exceeding 10 to avoid statistical bias and light spillover interference, which helps accurately depict real urbanized areas.


$$popindex=\frac{p}{a}=\frac{\frac{pop1000_{it}}{pop_{it}}\times 100\%}{\frac{area1000_{it}}{area_{it}}\times 100\%}$$ (1)

where:

*popindex* refers to the population agglomeration index;

*pop*1000_*it*_ represents the actual population;

*area*1000_*it*_ represents the grid area contained in the real urbanized area, that is, the actual utilization area;

*pop*_*it*_ refers to the population included in the entire metropolitan administrative area; and

*area*_*it*_ represents the sum of the areas of all the grids within the administrative division of the metropolitan area.

#### 3.2.2. Kernel density estimation.

This study employs the kernel density estimation method to analyze the dynamic evolution trends of population agglomeration in different metropolitan areas. The kernel density estimation method possesses favorable statistical properties, with a weaker dependence on models, and is widely applied in studies involving nonuniform spatial distributions. The density function of random variables *x*_1_, *x*_2_, *x*_3_... *x*_*i*_ is as shown in Equation (2).


$$f\left(x\right)=\frac{1}{Nh}\sum_{i=k}^{N}k\;\left(\frac{x_{i}-x}{h}\right)$$ (2)

Equation (2) represents the general formula for Kernel Density Estimation.

where:

f*(x)* represents the kernel density function of population concentration;

*N* denotes the number of observations;

*x*_*i*_ represents independently and identically distributed observations.

*k* represents the kernel function; and.

*h* signifies the smoothing parameter or bandwidth.

A larger h results in a smoother density curve, which may lead to greater estimation bias. Therefore, in this study, a smaller bandwidth with a smoother curve is chosen.


$$\{\begin{array}{c} lim\;k\left(x\right),x=0\\ \int_{-\infty }^{+\infty }k\left(x\right)dx=1,k\left(x\right)>0\\ \int_{-\infty }^{+\infty }k^{2}\left(x\right)dx<+\infty ,sup\;k\left(x\right)<+\infty \end{array}$$ (3)

where:

*x* represents independent and identically distributed observation values.

*k* represents the kernel function.

The kernel function is a smoothing transformation function that must satisfy the condition specified in Equation (3). Various kernel functions can be utilized, including the Gaussian kernel function, triangular kernel function, and box kernel function. The likelihood of selecting the Gaussian kernel function for estimation increases as the number of grouped data points decreases [35]. Therefore, in this study, the Gaussian kernel function is chosen to estimate the distribution dynamics and evolution trends of population concentration in different metropolitan areas.

#### 3.2.3. Markov chain model.

To overcome the limitation that kernel density estimation cannot describe the dynamics of the population agglomeration level distribution in detail, the Markov chain model was further used for analysis. The traditional Markov chain calculates the probability distribution and evolution trend of each type under the condition that the time and state are both discrete. This study explores the dynamic evolution characteristics of the population agglomeration level distribution in the Xi’an metropolitan area during different periods.


$$\left\{X\left(t\right),\;t\in M\right\}$$ (4)

where:

M refers to the exponential set of the random process corresponding to each period, and the finite state corresponds to the number of states of the random variable.

Then, Equation (4) is satisfied for all periods *t* and all possible states *j* and *i*.


$$\left\{X_{t}=j|X_{t-1}=i_{t-1},X_{t-2}=i_{t-2},\cdots X_{0}=i_{0}\right\}=P\left\{X_{t}=j|X_{t-1}=i\right\}$$ (5)

where:

Equation (5) expresses the properties of the first-order Markov chain, that is, the probability that the random variable *X* is in state *j* at period *t* only depends on the state of *X* at period *t* − 1.


$$P_{ij}=\frac{n_{ij}}{n_{j}}$$ (6)

Assume that the state space of the Markov process is *I*, where *I* = {1,2,3,...}. The matrix *P* = *P*_*ij*_, where *i* , *j* ∈ I represents the state transition probability matrix when the process changes from state *i* to state *j*. The matrix composed of all transition probabilities *P*_*ij*_ is called the state transition probability matrix (Table 1). This probability can be estimated according to Equation (6).

**Table 1:** Markov transition probability matrix (k = 4).

t/t + 1	1	2	3	4
1	*P*_11_	*P*_12_	*P*_13_	*P*_14_
2	*P*_21_	*P*_22_	*P*_23_	*P*_24_
3	*P*_31_	*P*_32_	*P*_33_	*P*_34_
4	*P*_41_	*P*_42_	*P*_43_	*P*_44_

*n*_*ij*_ is the total number of times the state of the observed object transitions from state *i* in year *t* to state *j* in year *t + 1* during the examination period.

*n*_*j*_ is the total number of occurrences of state *j* in the initial period.

#### 3.2.4. Convergence model.

(1) α Convergence model

This study refers to whether the gap in population agglomeration levels in metropolitan areas decreases over time, measured via the coefficient of variation. If there is a trend of decreasing population concentration levels in the seven major metropolitan areas, it signifies the presence of α convergence. The specific formula is as follows.


$$K=\sqrt{{\sum_{i}^{n}\left(P_{i}-\bar{P}\right)}^{2}/n}\;/\bar{P}$$ (7)

where:

*i* represents the *i*-th metropolitan area (i = 1,2,3, …, N);

*n* denotes the total number of metropolitan areas;

*P*_*i*_ signifies the population concentration level of the *i*-th metropolitan area;

$\bar{P}$ represents the mean population concentration level; and

*K* represents the coefficient of variation.

If the *K* value gradually decreases during the sample period, it indicates the presence of α-convergence in population agglomeration levels.

(2) β Convergence model

β convergence methods exhibit a high degree of quantification and are derived from economic convergence theory in neoclassical economics. They are categorized into absolute β convergence and conditional β convergence. This study refers to the phenomenon where over time, metropolitan agglomerations with lower population concentrations experience higher growth rates, ultimately catching up with metropolitan agglomerations with higher population concentrations and converging to a stable level. This study employs both absolute β convergence models and relative β convergence models to analyze the convergence characteristics of population concentration in various metropolitan agglomerations over time. The panel data β convergence model used in this study is as follows.


$$\ln\frac{P_{it}}{P_{it-1}}=a+\beta \ln P_{it-1}+\mu_{it}+e_{it}$$ (8)

$$\ln\frac{P_{it}}{P_{it-1}}=a+\beta \ln P_{it-1}+\gamma \ln Z_{it-1}+\mu_{it}+e_{it}$$ (9)

where:

*P*_*it*_ and *P*_*it* − 1_ represent the population concentration levels at the beginning and end of each metropolitan agglomeration’s respective period.

*Z*_*it* − 1_ is the control variable group, which includes public financial expenditure (PFE), economic development level (ECO) and basic education (BE);

γ represents the estimated coefficient for the control variable;

*e*_*it*_ is the random disturbance term; and

*μ*_*it*_ represents the potential fixed effects or random effects in panel data, following a normal distribution $N\left(0,\;\sigma^{2}\right)$.

If *P*_*it*_ exhibits β convergence, the β coefficient is negative, indicating the presence of convergence among different metropolitan agglomeration population concentration levels. If it is positive, it suggests divergence.

### 3.3. Variable description

Academic Translation: In this study, the population concentration index, calculated according to Equation (1), is used to measure the population concentration levels in different metropolitan agglomerations. This study draws inspiration from the research of Yang [36]. The control variables of this conditional β convergence model include variables such as public financial expenditure, economic development level and basic education.

The economic development level (ECO) is measured using the total GDP of each metropolitan agglomeration. Numerous studies suggest that economically developed regions possess a stronger population “pull” capacity, making regions with higher economic development levels more conducive to population concentration. Public fiscal expenditure (PFE) is quantified by the total amount of public fiscal expenditure within each metropolitan agglomeration. Public fiscal expenditure affects the level of public service provision, subsequently influencing population agglomeration in that area. Basic education (BE) is measured by the total number of primary and secondary school students in each region within the metropolitan area. High-quality educational resources are an important driving force for attracting population flow to cities and are conducive to population agglomeration.

## 4. Dynamic evolution and convergence analysis of nonequilibrium population agglomeration in the Xi’an metropolitan area of China

### 4.1. Kernel density estimation results

**Fig 1:**
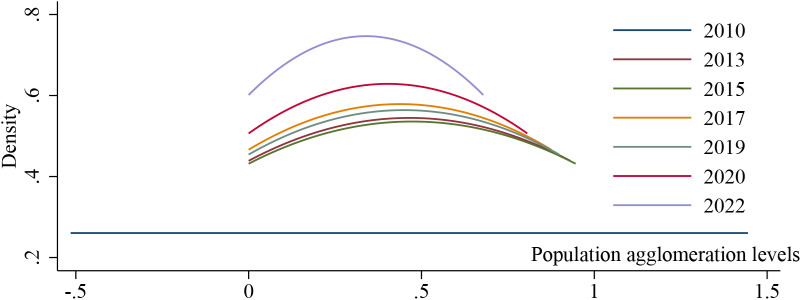
Kernel density estimation of Xi’an metropolitan agglomeration.

To analyze the dynamic evolution of population agglomeration in the Xi’an metropolitan area, this paper uses kernel density estimation to analyze the distribution position, ductility and polarization trend of population agglomeration in the Xi’an metropolitan area during the sample period. The estimation results are illustrated in Fig 1. The kernel density curve reveals the following insights. First, in terms of distribution, the central position of the curve initially shifts to the right and later to the left, indicating an initial increase followed by a decrease in population agglomeration levels within the Xi’an metropolitan agglomeration. Second, regarding the shape of the distribution, the kernel density curve, on the whole, reveals an increase in the height of the main peak and a reduction in the overall width, suggesting a decrease in the absolute differences in population agglomeration levels within the Xi’an metropolitan agglomeration during the sample period. This shows that there is no phenomenon in which the population agglomeration level of some cities in the Xi’an metropolitan area is much higher than that of other cities. For example, that of Xi’an City is higher than that of the Linwei District of Weinan City. Fourth, from the perspective of the polarization phenomenon, the core density curve of population agglomeration in the Xi’an metropolitan area shows an obvious bimodal distribution, which indicates that there is no obvious polarization phenomenon in the population agglomeration in the Xi’an metropolitan area. The analysis of the kernel density curve reveals that the population agglomeration level of the Xi’an metropolitan area shows a trend of first increasing and then decreasing. There is no excessive gap between the population agglomeration levels of two cities within the region, and there is no polarization phenomenon.

**Fig 2:**
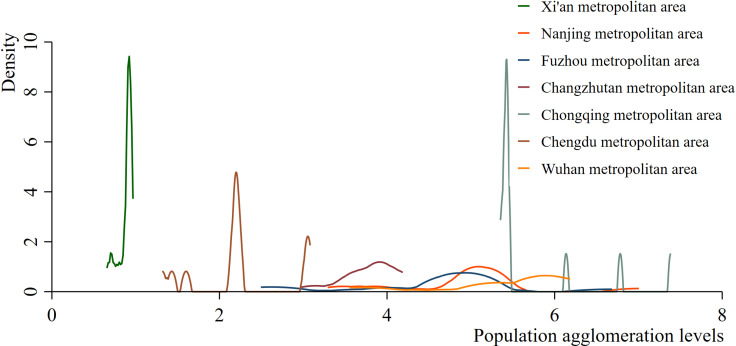
Comparison of Kernel density estimations between Xi’an metropolitan agglomeration and other metropolitan agglomerations officially approved by the National Development and Reform Commission (NDRC).

A further analysis of the uneven dynamic evolution differences between the Xi’an metropolitan agglomeration and other metropolitan agglomerations officially approved by the National Development and Reform Commission (NDRC) is presented in Fig 2. Compared with those of metropolitan agglomerations such as Nanjing, Wuhan, Fuzhou, Changzhutan, Chongqing, and Chengdu, the kernel density curve for the Xi’an metropolitan agglomeration is steeper, with the central peak of the kernel density curve noticeably shifting to the left. This indicates a significant disparity in population agglomeration levels between the Xi’an metropolitan agglomeration and other metropolitan agglomerations. In addition, the comparative kernel density map of urban agglomerations during the survey period reveal that the Fuzhou urban agglomeration and the Chengdu urban agglomeration has relatively obvious multipeak distributions, indicating that there is an obvious polarization in the population concentration levels of the Fuzhou urban agglomeration and the Chengdu urban agglomeration. The Chongqing, Nanjing, and Fuzhou metropolitan agglomerations display distinct right-skewed tail phenomena, indicating that within these regions, certain cities experience significantly higher population agglomeration levels than others do. Through comparison, it was found that there was a large gap between the population agglomeration level of the Xi’an metropolitan area and that of other metropolitan areas during the sample period. However, there is no uneven development of population agglomeration within the Xi’an metropolitan area. During the sample period, the Fuzhou metropolitan area, Nanjing metropolitan area, Chengdu metropolitan area, and Chongqing metropolitan area all experienced uneven development in terms of population agglomeration levels, whereas there was no imbalance in population development within the Wuhan or Changzhutan metropolitan areas.

### 4.2. Markov chain model estimation results

Although the kernel density curve depicts the overall shape and dynamic evolution of the population agglomeration level distribution in a metropolitan area, it cannot reflect its long-term evolution trend. This study further uses the traditional Markov chain model to predict the evolution trend of population agglomeration levels in different metropolitan areas. First, the population agglomeration level is discretely divided into four levels according to the quartile method, namely, the low level (I), medium-low level (II), medium-high level (III) and high level (IV). The Markov chain transition probability matrix of the population agglomeration level is subsequently calculated with a lag of one year, and the results are shown in Table 2.

**Table 2:** Markov transition probability matrix (k = 4).

t/t + 1	1	2	3	4
1	0.6250	0.3125	0.0625	0.0000
2	0.2308	0.5385	0.2308	0.0000
3	0.0625	0.1250	0.6875	0.1250
4	0.2000	0.0000	0.0667	0.7333

The results in Table 2 show that the population agglomeration level of 62.5% of the metropolitan areas remains at a low level and that the population agglomeration level of 31.25% of the metropolitan areas shifts upward to the medium-low level after one year. The probabilities that the population agglomeration level will jump forward to the medium-high and high levels are 6.25% and 0%, respectively.

The probability that the population agglomeration level will remain unchanged at a medium or low level one year later is 53.85%. The probability that the population agglomeration level will shift upward to the medium-high level is 23.08%. The probability that the level of population agglomeration will jump forward to the high level is 0%, and the probability of it shifting to the low level is 23.08%.

The population agglomeration level of 68.75% of the metropolitan areas will still be medium-high one year later. A total of 12.50% of the metropolitan population agglomeration level will shift upward to the high level. The probability of the population agglomeration level shifting downward to the medium-low level is 12.50%, and the probability of it jumping to the low level is 6.25%.

There is a 73.33% probability that the population agglomeration level will remain at the high level one year later. The probability that the population agglomeration level will shift downward to the medium-high level is 6.67%. The probabilities of a negative jump in the population agglomeration level to the medium-low level and low level are 0% and 20%, respectively.

The above analysis results indicate that in the Markov transition probability matrix at the population agglomeration level, the transition probabilities on the diagonal are much greater than the probabilities on the off-diagonal. That is, when the probability of maintaining the original level is greater than the transition probability, clubs at that level will converge. Therefore, there is an obvious “club convergence” phenomenon at the level of population agglomeration, and the probabilities of medium-high level convergence and high-level convergence are relatively large, 68.75% and 73.33%, respectively. The probabilities of low-medium level and low-level convergence are smaller, 53.85% and 62.5%, respectively. That is, the probability that the level of population agglomeration remains stable is at least 53.85%, indicating that there is a “Matthew effect” at the level of population agglomeration in which the rich become richer and the poor become poorer.

## 5. An empirical analysis of the convergence of population agglomeration in Xi’an metropolitan area of China

### 5.1. α Convergence

This study tests the α convergence of the population agglomeration level in the Xi’an metropolitan area by measuring the coefficient of variation of the population agglomeration level in the Xi’an metropolitan area from 2010 to 2022. The coefficient of variation of population agglomeration between the Xi’an metropolitan area and the Nanjing, Fuzhou, Wuhan, Changzhutan, Chongqing, and Chengdu metropolitan areas was measured to test its α convergence characteristics. The results are shown in Table 3. The coefficients of variation of the Xi’an metropolitan area and the Xi’an-Nanjing metropolitan area generally shows an upward and then downward trend, and the α convergence characteristics are not obvious. For the Xi’an-Fuzhou metropolitan agglomeration, the coefficient of variation displays an overall trend of initially decreasing, then increasing, followed by another decrease, with no clear α convergence characteristics observed. The Xi’an-Wuhan metropolitan agglomeration’s coefficient of variation exhibits an overall trend of initially increasing, then decreasing, followed by an increase, with no significant α convergence characteristics observed. Before 2017, the coefficient of variation for the Xi’an–Changzhutan metropolitan agglomeration noticeably increases, indicating a clear divergence in population concentration levels between the two regions. Starting in 2018, there is a slight decrease in the coefficient of variation, indicating a minor α convergence phenomenon. The coefficient of variation of the Xi’an–Chongqing metropolitan area generally shows a trend of first increasing, then decreasing, and then increasing again, and the α convergence characteristics are not obvious. For the Xi’an–Chengdu metropolitan agglomeration, the coefficient of variation increases from 2010 to 2012, decreases from 2013 to 2016, and then increases again in 2017 before continuing to decrease, with no apparent α convergence characteristics observed.

**Table 3:** α convergence of Xi’an metropolitan area and other metropolitan areas officially approved by the National Development and Reform Commission.

Year	Xi’an	Xi’an-Nanjing	Xi’an-Fuzhou	Xi’an-Wuhan	Xi’an-Changzhutan	Xi’an-Chongqing	Xi’an-Chengdu
2010	0.9296	0.9900	1.0689	1.0007	0.8383	0.9957	0.7501
2011	0.9296	0.9482	0.9861	1.0043	0.8396	0.9966	0.7541
2012	0.9302	0.9708	0.9570	1.0052	0.8514	1.0019	0.7585
2013	0.9304	0.9736	0.9596	1.0057	0.8593	1.0015	0.5800
2014	0.9237	0.9803	0.9633	1.0450	0.8718	1.0024	0.5852
2015	0.9459	0.9726	0.9029	1.0384	0.8747	0.9932	0.5731
2016	0.9351	0.9791	0.9619	1.0425	0.8873	0.9964	0.5585
2017	0.8755	1.0086	0.9876	1.0635	0.9213	1.0208	0.6011
2018	0.8901	1.0023	0.9832	1.0579	0.9147	1.0151	0.5956
2019	0.8986	1.0926	0.9793	1.0544	0.9145	1.0134	0.5939
2020	0.8060	0.9426	0.9158	1.0084	0.9108	1.0859	0.4674
2021	0.7350	0.9414	0.8159	0.9295	0.9019	1.1377	0.4007
2022	0.6788	0.9307	0.8091	0.9861	0.8889	1.1761	0.5041

Note: ***, **, and *  indicate significance at the 1%, 5%, and 10% levels respectively.

The analysis reveals that only the level of population agglomeration between the Xi’an-Changzhutan Metropolitan District has slight α convergence characteristics and that there are no α convergence characteristics between the Xi’an Metropolitan Area and other metropolitan areas. The level of population agglomeration is affected by various factors. Possible economic factors include the basic living conditions of income and employment opportunities, local economic development factors, and the size of the gap between the rich and the poor.

### 5.2. β convergence results

#### 5.2.1. Absolute β convergence.

To further characterize the evolution trend of population agglomeration levels accurately between the Xi’an-Nanjing, Xi’an-Fuzhou, Xi’an-Wuhan, Xi’an-Changzhutan, Xi’an-Chongqing, and Xi’an-Chengdu metropolitan areas, the absolute β convergence equations of the population agglomeration levels of Xi’an-Nanjing, Xi’an-Fuzhou, Xi’an-Wuhan, Xi’an-Changzhutan, Xi’an-Chongqing, and Xi’an-Chengdu are constructed and analyzed via fixed effects regression models. The regression results are shown in Table 4. The absolute convergence coefficient β is positive, with Xi’an-Nanjing, Xi’an-Fuzhou, Xi’an-Wuhan, Xi’an-Changzhutan, and Xi’an-Chongqing being significant at the 1% level and Xi’an-Chengdu at the 5% level. This signifies a notable divergence in population concentration levels between the Xi’an metropolitan agglomeration and the Nanjing, Fuzhou, Wuhan, Changzhutan, Chongqing, and Chengdu metropolitan agglomerations, with no evidence of absolute β convergence. This suggests that the population concentration gap between the Xi’an metropolitan agglomeration and other metropolitan agglomerations is continuously widening.

**Table 4:** Absolute β convergence results.

Variables	Xi’an-Nanjing	Xi’an-Fuzhou	Xi’an-Wuhan	Xi’an- Changzhutan	Xi’an-Chongqing	Xi’an-Chengdu
	FE	FE	FE	FE	FE	FE
	lnpopindex	Lnpopindex	lnpopindex	lnpopindex	Lnpopindex	lnpopindex
β (lnpopindex-1)	0.5941^***^	0.4428^**^	0.7718^***^	0.6838^**^	1.0108^***^	0.7672^**^
Constant	0.7788^***^	0.744^***^	0.8409^***^	0.6395^***^	0.8018^***^	0.3919^***^
R-squared	0.7962	0.3107	0.5987	0.3391	0.8293	0.3255

Note: ***, **, and *  indicate significance at the 1%, 5%, and 10% levels respectively.

#### 5.2.2. Conditional β convergence.

To test whether conditional β convergence exists in population concentration levels between the Xi’an-Nanjing, Xi’an-Fuzhou, Xi’an-Wuhan, Xi’an-Chanzhutan, Xi’an-Chongqing, and Xi’an-Chengdu metropolitan agglomerations, considering the influence of economic development, public fiscal expenditure, and basic education factors, conditional β convergence equations are separately constructed for these metropolitan agglomerations and subjected to analysis. The results are presented in Table 5. The fixed effects regression results show that after the factors of economic development level, public fiscal expenditure and basic education are introduced, they are still significantly positive, indicating that there are significant differences between the Xi’an metropolitan area and the Nanjing metropolitan area. There are significant divergence characteristics among them, they do not tend to converge at the same speed over time, and the gap in population agglomeration levels continues to increase. For the Xi’an-Fuzhou and Xi’an-Chongqing metropolitan agglomerations, the results of the fixed effects regression models indicate that, considering economic development, public fiscal expenditure, and basic education factors, the β parameter is significantly positive. This suggests that there is no convergence in population concentration levels between the Xi’an metropolitan agglomeration and the Fuzhou metropolitan agglomeration or between the Xi’an metropolitan agglomeration and the Chongqing metropolitan agglomeration. Instead, there are clear characteristics of divergence. For the Xi’an-Wuhan, Xi’an-Changzhutan, and Xi’an-Chengdu metropolitan agglomerations, both fixed effects and random effects regression tests were conducted to examine their β convergence characteristics. The results show that the β parameter is significantly positive in all cases, indicating the presence of divergence in population agglomeration levels between the Xi’an metropolitan agglomeration and the Wuhan metropolitan agglomeration, the Changzhutan metropolitan agglomeration, and the Chengdu metropolitan agglomeration.

**Table 5:** Conditional β convergence results.

Variables	Xi’an-Nanjing	Xi’an-Fuzhou	Xi’an-Wuhan	Xi’an-Changzhutan	Xi’an-Chongqing	Xi’an-Chengdu
	FE	FE	FE	FE	FE	FE
	lnpopindex	Lnpopindex	lnpopindex	lnpopindex	Lnpopindex	lnpopindex
β (lnpopindex-1)	0.5941^***^	0.4428^**^	0.7718^***^	0.6838^**^	1.0108^***^	0.7672^**^
Constant	0.7788^***^	0.744^***^	0.8409^***^	0.6395^***^	0.8018^***^	0.3919^***^
R-squared	0.7962	0.3107	0.5987	0.3391	0.8293	0.3255

Note: ***, **, and *  indicate significance at the 1%, 5%, and 10% levels respectively.

In summary, with the use of the β convergence model for convergence analysis, the results show that the absolute β convergence and conditions β convergence indicate that the gap between the population agglomeration level between the Xi’an metropolitan area and other metropolitan areas has continued to expand. Owing to the obvious level of economic development between cities and different industrial structure distributions, there are also differences in the rainbow absorption effects of populations of different levels. The proportion of price levels and wage income between regions may be among the economic factors influencing the choice of population migration.

## 6. Analysis of the motivations of population agglomeration in Xi’an metropolitan area

### 6.1. Construction of an integrated theoretical framework for the driving forces of population agglomeration

Compared with the Nanjing, Fuzhou, Wuhan, Changsha-Zhuzhou-Xiangtan, Chongqing, and Chengdu metropolitan areas, Xi’an, as an important node city on the Silk Road, has greater development potential, especially after the “One Belt, One Road” strategy was proposed in 2013. The Xi’an Metropolitan Area has become the strategic fulcrum of “Belt and Road” construction, and its population agglomeration level has also rapidly improved. However, there are still many obstacles to the development of population agglomeration. Therefore, this section, which measures the population agglomeration level of the Xi’an metropolitan area and analyzes its dynamic evolution characteristics and convergence differences with other metropolitan areas, further explores the reasons leading to differences in population agglomeration levels by constructing an integrative theoretical framework for population agglomeration research.

**Fig 3:**
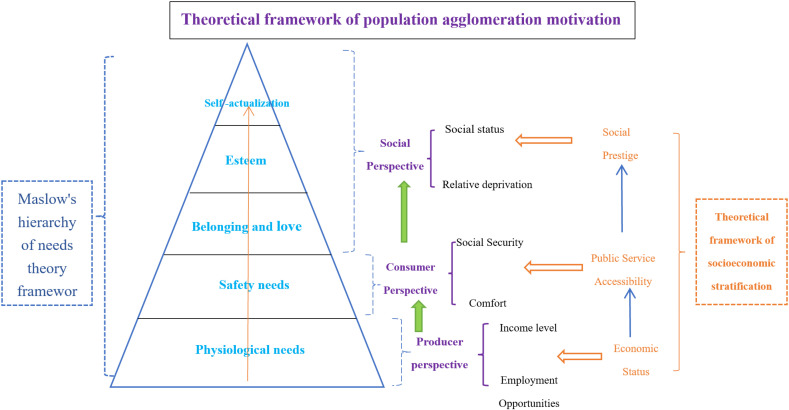
Framework for the analysis of population agglomeration constraints.

On the basis of Maslow’s hierarchy of needs theory, and drawing on the three-dimensional socioeconomic stratification theoretical framework proposed by Qi and Wang [37] for China’s floating population and the three perspectives of labor force migration motivation proposed by Wang [31], we correspond the five levels of needs and the three perspectives of population agglomeration motivation to the three-dimensional socioeconomic stratification theoretical framework. Starting from the perspectives of producers, consumers, and social people, we construct an integrated theoretical framework of population agglomeration motivation, and analyze the motivations that lead to the differences in population agglomeration levels between the Xi’an metropolitan area and other metropolitan areas, as shown in Fig 3.

The framework diagram of population agglomeration motivations shows that, from the “producer perspective”, it mainly includes income level and employment opportunity factors, which reflect the economic status of the migrant population in the socioeconomic stratification and correspond to the most basic physiological needs factors in Maslow’s hierarchy of needs. This group of people mainly considers survival as the basic goal. The development of urban agglomerations can provide the most basic living security to meet their needs and attract the migrant population with such needs. From the perspective of the “consumer”, after meeting the basic conditions of the “producer perspective”, the floating population will pursue a more comfortable living environment, including both a comfortable material environment and a public service environment. This is consistent with the population’s pursuit of security needs in Maslow’s hierarchy of needs theory and reflects the importance that the floating population attaches to the degree of access to public services in socioeconomic stratification theory. From the perspective of social people, whether the floating population has a sense of relative deprivation and whether they can obtain social status in the local area are important motivators for their migration behavior. This is consistent with the purpose of the floating population in the socioeconomic stratification to gain social prestige. From the perspective of Maslow’s hierarchy of needs, the main needs of the floating population in this class are to obtain belonging, love and respect and to be able to realize themselves.

### 6.2. Empirical analysis

#### 6.2.1. Sample selection and data sources.

This section selects China’s Xi’an Metropolitan Area, Nanjing Metropolitan Area, Fuzhou Metropolitan Area, Wuhan Metropolitan Area, Changzhutan Metropolitan Area, Chongqing Metropolitan Area, and Chengdu Metropolitan Area as the research objects, and the specific zoning range is the same as that described above. The data interval is selected from 2010 to 2022. The data mainly come from the China Statistical Yearbook, the Population Statistical Yearbook, the City Statistical Yearbook, the Guotai An Database, the Wind Database, the Provincial Statistical Yearbook, the District and County Statistical Yearbook and the Regional Development Bulletin. The interpolation method is used to supplement the missing data. Since some district and county yearbook data are only updated to 2022, to unify the data caliber, the data in this section are selected up to 2022.

#### 6.2.2. Variable selection and model building.

(1) Variable selection

Based on the analysis of the above theoretical model, starting from three dimensions, this paper empirically analyzes the reasons for the differences in population agglomeration levels between the Xi’an metropolitan area and other metropolitan areas. Drawing on the research of Wang [31], this study selects income level and employment opportunity indicators from the producer dimension and selects education level, public consumption expenditure, and natural comfort indicators from the consumer dimension. Drawing on the research of Qi and Wang [37], since education level is both a source of social stratification and an important factor affecting socioeconomic differences, this study selects education level indicators from the “social person” dimension.

The income level is measured by the per capita disposable income in urban areas, employment opportunities are measured by the labor participation rate, the education level is measured by the number of students in primary and secondary schools, public consumption expenditure is measured by public fiscal expenditures, natural comfort is measured by the temperature and humidity index, and the education level is measured by the average years of education.

The calculation method of the temperature and humidity indices in the “Evaluation of Climate Comfort of the Human Residential Environment” is used to calculate the temperature and humidity indices. The calculation is as follows.


$$I=T-0.55\times \left(1-RH\right)\times \left(T-14.4\right)$$ (10)

where:

*I* represents the temperature and humidity index;

*T* represents the average temperature during a certain evaluation period, in degrees Celsius (°C); and

*RH* represents the average relative humidity during a certain evaluation period.

Referring to previous studies [38], the measurement and calculation of the average years of education are as follows.


$$AYD=\;\left(Np\;*\;6\;+\;NJH\;*9\;+\;NHT\;*\;12\;+\;NCB\;*\;16\right)/TP$$ (11)

where:

*AYD* represents the average years of education;

*NP* represents the number of people with a primary school education;

*NJH* represents the number of people with junior high school education;

*NHT* represents the number of people with high school and technical secondary school education; *NCB* represents the number of people with a college and bachelor degree or above; and

*TP* represents the total population over 6 years old.

(2) Panel regression model

On the basis of the research of Xu et al. [39], this study constructs a multivariate regression model to analyze the causes of population agglomeration differences. The designed regression model is as follows.


$$popindex=\alpha_{0}+\alpha_{1}RJZP+\alpha_{2}LDCY+\alpha_{3}PFE+\alpha_{4}BE+\alpha_{5}WS+\alpha_{6}AYD+\xi$$ (12)

where:

*α*_0_ is a constant term;

*α*_1_ 、 *α*_2_ 、 *α*_3_ 、 *α*_4_ 、 *α*_5_ 、 *α*_6_ are regression coefficients;

*ξ* is a random variable;

*RJZP* represents the per capita disposable income of urban residents;

*LDCY* represents the per capita disposable income;

*PFE* represents public fiscal expenditure;

*BE* represents the number of students in primary and secondary schools;

*WS* represents the temperature and humidity index; and

*AYD* represents the average years of education.

(3) Geographic detector

As a spatial statistical method, factor detection by a geographic detector can measure the factor effect. By using this method to calculate the *q* value of each agglomeration factor, we can explain the spatial variability of population agglomeration between the Xi’an metropolitan area and other metropolitan areas and perform inference statistics on their distribution patterns.

The principle of factor detection is that the independent variable and the dependent variable have their own spatial distribution characteristics. If the two are similar, the independent variable has an important influence on the dependent variable. Using the factor detection module of the geographic detector, we can test whether a factor has explanatory power for the spatial distribution difference of a certain explained variable. Therefore, this study uses factor detection to test the explanatory effect of each factor on the spatial distribution pattern of the difference in the level of population agglomeration in the metropolitan area. The calculation formula is as follows.


$$q=1-\frac{\sum_{h=1}^{L}\;N_{h}\delta_{h}^{2}}{N\delta^{2}}$$ (13)

where:

*q* is the detection value of the detection factor *x* on population agglomeration.

*L* is the stratification of the influencing factor *x*;

*N*_*h*_ and *N* are the total number of samples in layer *h* and the study area respectively; and

$\delta_{h}^{2}$ and *δ*^2^ are the variances of layer *h* and the study area, respectively.

The value range of *q* is [0, 1]. The larger the *q* value is, the stronger the explanatory power of the detection factor X on the dependent variable, and vice versa.

#### 6.2.3. Analysis of the empirical results.

Judging from the regression results in Column (1) of Table 6, the R-squared value of the model is 0.747, indicating that the model has a good fit. The F statistic of the model is 41.36, indicating that the model as a whole has passed the significance test and that a linear relationship is established. The regression results show that the coefficient of *LDCY*, the producer dimension indicator, is 0.195, but it does not pass the significance test, indicating that the regression results cannot confirm the positive impact of employment opportunities on population agglomeration. On the basis of previous research on the influencing factors of population agglomeration, the labor participation rate significantly promotes population agglomeration. The reason for the above results is that there is a lag effect in the impact of the labor participation rate on population agglomeration. That is, the floating population will decide whether to migrate in the next year on the basis of the number of employment opportunities in the place of immigration that year.

**Table 6:** Regression results.

Variables	(1)	(2)
	popindex	q-value
lnRJZP	−3.697***	0.2102***
	(−7.01)	
lnLDCY	0.195	0.2523***
	(0.54)	
lnPFE	1.211***	0.2730***
	(4.36)	
lnBE	0.479***	0.4698***
	(3.26)	
lnWS	13.810***	0.5884***
	(12.81)	
lnAYD	7.482***	0.2102***
	(3.23)	
Constant	−28.857***	
	(−4.80)	
Observations	91	
R-squared	0.747	
F-statistic	41.36	

Note: The values in parentheses are t. ***, ** and *  indicate significance at the 1%, 5% and 10% levels respectively.

The *RJZP* coefficient is −3.697 and passes the 1% significance test, indicating that urban per capita disposable income has a significant negative effect on the population agglomeration level of sample urban areas. This is partly related to the large number of rural people flowing into cities. The low income of rural migrants leads to a reduction in the per capita disposable income level in local cities and towns.

For consumer dimension indicators, the coefficients of *PFE*, *BE*, and *WS* are all positive at 1.211, 0.479, and 13.810, respectively, and all pass the 1% significance test. This shows that public consumption expenditures, the education level and natural comfort significantly attract population agglomeration and are important drivers of the difference in population agglomeration levels between the Xi’an metropolitan area and other metropolitan areas.

For the social person dimension index, the coefficient of *AYD* is 7.482 and passes the 1% significance test. This shows that educational level significantly affects population agglomeration and is an important factor in the population agglomeration level of the Xi’an metropolitan area and other metropolitan areas.

#### 6.2.4. Further analysis.

The geographic detector method is further used to test whether the influencing factors of each dimension indicator have explanatory power for the spatial distribution differences in population agglomeration levels of different metropolitan areas. The results are shown in Column (2) of Table 6. According to the factor detection results, the five driving factors are all significant at the 1% level, indicating that they have a strong ability to explain the spatial differentiation of population agglomeration in China’s urban areas.

The *q* values of *RJZP* and *LDCY* are 0.2102 and 0.2523, respectively, which explains the motivation for the difference in population agglomeration levels in urban agglomerations from the perspective of producers. Employment opportunities explain population agglomeration more than employment income does. According to the explanation of the motivation of population migration in neoclassical economics, the wage difference between the two labor markets will guide the labor force from the low-wage market to the high-wage market. The existence of a gap between the income level and employment opportunities will prompt China’s working-age migrant population to pursue higher economic income and better employment opportunities and make decisions to move across regions.

The *q* values of *PFE*, *BE*, and *WS* are 0.2730, 0.4698, and 0.5884, respectively. They explain the motivation for population agglomeration differences from the perspective of consumers. Natural comfort has the greatest explanatory power for the motivation of population agglomeration, followed by education level and public consumption expenditure. Among the consumer dimension indicators, natural comfort has the greatest appeal to population agglomeration, indicating that migrant populations are more inclined to migrate to areas with a comfortable climate and suitable living conditions. The second is the local education level, indicating that migrant populations consider the environment in which their children receive an education when they gather.

By comprehensively considering the three dimensions, the q values of each indicator are ranked as follows.

WS>BE>PFE>LDCY>AYD=RJZP (0.5884>0.4698>0.2730>0.2523>0.2102=0.2102).

This shows that natural comfort has the strongest explanatory power for the spatial differentiation of population agglomeration, followed by the education level, public consumption expenditures, and employment opportunities. The income level and education level have the same explanatory power. The consumer dimension indicators have the strongest explanatory power for the spatial differentiation of population agglomeration, which shows that the main driving force of population agglomeration is factors in the consumer dimension.

## 7. Discussion

The existing research has focused mainly on the influencing factors of a certain dimension of population agglomeration. Most of the traditional indicators, such as the population density, are used to measure the level of population agglomeration. This study combines nighttime light data with population density data to construct a population agglomeration index to measure the level of population agglomeration. This study specifically conducts targeted regional research aimed at the population agglomeration of the Xi’an metropolitan circle of the 13th dynasty in China and analyzes its dynamic evolution characteristics of population agglomeration. Second, research on the factors of population agglomeration has focused on specific aspects of the economy and consumption. There are few studies that include three aspects—economic, consumption and social factors—into the same social flow framework and conduct integrated analyses of the cause of population agglomeration. Therefore, this study constructs an integrated theoretical framework of population agglomeration based on socioeconomic stratification theory, Maslow’s hierarchy of needs theory and the social mobility perspective. This study empirically analyzes the causes of the differences in population agglomeration between the Xi’an metropolitan area and other metropolitan areas in three dimensions: producers, consumers and social people.

Compared with other metropolitan areas, the population agglomeration level of the Xi’an metropolitan area is quite different from that of other metropolitan areas, and the β convergence analysis reveals that the difference between the population agglomeration levels of the Xi’an metropolitan area and other metropolitan areas gradually increases over time. The results of the analysis of the population agglomeration drivers reveal that the producer dimension indicator income level, the consumer dimension indicator natural comfort, the education level, public consumption expenditures, and the social people dimension indicator education level are all significant drivers of population agglomeration. Only the employment opportunity indicator in the producer dimension is not significant, which is partly related to the existence of a lag effect; that is, the floating population will make a decision on whether to migrate in the next year based on the number of employment opportunities in the place of inflow that year. The geodetector factor detection results reveal that consumer dimension indicators explain the driving force of population agglomeration the most, so investing in consumer dimension indicators is more likely to attract population agglomeration.

This study establishes an integrated framework for population agglomeration research to provide a theoretical framework and ideas for developing countries to analyze urban population agglomeration, as well as further expands the literature and provides relevant cases for the study of population agglomeration. It also provides policy-making ideas for promoting population development in developing countries, promoting population development, attracting population agglomeration, and further promoting urban and economic development.

## 8. Conclusions and countermeasures

### 8.1. Conclusions

Kernel density estimation was used to analyze the dynamic evolution trend of population agglomeration in the Xi’an metropolitan area from 2010 to 2022. The results revealed that the population agglomeration level of the Xi’an metropolitan area first increased but then decreased. During the sample period, the absolute gap in population agglomeration levels within the Xi’an metropolitan area gradually decreased. There is no phenomenon in the Xi’an metropolitan area where the population agglomeration level of some cities is much higher than that of other cities. There is no obvious polarization phenomenon in the population agglomeration of the Xi’an metropolitan area. Compared with those of the Nanjing, Wuhan, Fuzhou, Changzhutan, Chongqing, and Chengdu metropolitan areas, the population agglomeration level of the Xi’an metropolitan area is lower, and there is a large gap between them. In the Markov transition probability matrix at the population agglomeration level, the transition probabilities on the diagonal are much greater than those on the off-diagonal. That is, when the probability of maintaining the original level is greater than the transition probability, clubs at that level will converge. The probability that the level of population agglomeration remains stable is at least 53.85%, indicating that there is a “Matthew effect” at the level of population agglomeration in which the rich become richer and the poor become poorer.

By measuring the coefficient of variation of the population agglomeration level in the Xi’an metropolitan area from 2010 to 2022, the α convergence of the population agglomeration level in the Xi’an metropolitan area was tested. We also measured the variation coefficient of population agglomeration between the Xi’an metropolitan area and the Nanjing, Fuzhou, Wuhan, Changzhutan, Chengdu, and Chongqing metropolitan areas to test its α convergence characteristics. The α convergence characteristics of the Xi’an Metropolitan Area, Xi’an-Nanjing Metropolitan Area, Xi’an-Chongqing Metropolitan Area, Xi’an-Fuzhou Metropolitan Area, Xi’an-Chengdu Metropolitan Area, and Xi’an-Wuhan Metropolitan Area are not obvious. The population agglomeration level between the Xi’an and Changzhutan metropolitan areas has obvious characteristics of first diverging and then converging. The absolute β convergence equation and the conditional β convergence equation of the population agglomeration levels between the Xi’an-Nanjing, Xi’an-Fuzhou, Xi’an-Wuhan, Xi’an-Changzhutan, Xi’an-Chongqing, and Xi’an-Chengdu metropolitan areas were respectively constructed for analysis. The results show that there are significant differences in population agglomeration levels among the Xi’an-Nanjing, Xi’an-Fuzhou, Xi’an-Wuhan, Xi’an-Changzhutan, Xi’an-Chongqing, and Xi’an-Chengdu metropolitan areas, and they do not tend to trend over time. At the same convergence rate, the gap in population agglomeration levels continues to increase.

Based on Maslow’s hierarchy of needs theory, the theoretical framework of socioeconomic stratification and the three perspectives of labor migration motivation, the five levels of needs and the three perspectives of population agglomeration motivation are matched with the three-dimensional socioeconomic stratification theoretical framework. From the perspectives of producers, consumers and social people, an integrated theoretical framework of population agglomeration motivation is constructed, and the motivations leading to the difference in population agglomeration levels between the Xi’an metropolitan area and other metropolitan areas are empirically analyzed. The results show that the income level, natural comfort, education level, public consumption expenditures, and education level are all significant drivers of population agglomeration. Only the employment opportunity indicator in the producer dimension is not significant, which is partly related to the existence of a lag effect; that is, the floating population will make a decision on whether to migrate in the next year based on the number of employment opportunities in the place of inflow that year. The geodetector factor detection results reveal that consumer dimension indicators explain the driving force of population agglomeration the most, so investing in consumer dimension indicators is more likely to attract population agglomeration.

### 8.2. Countermeasures

The metropolitan area is an advanced spatial form of metropolitan development that can promote metropolitan modernization and realize new metropolitan areas. According to the development planning goals of the Xi’an metropolitan area, a modern Xi’an metropolitan area is planned by 2035. The metropolitan and global integration within the circle will be essentially realized, forming a new pattern of coordinated development of large, medium and small cities and small towns. On this basis, this article proposes the following countermeasures.

First, this study proposes targeted countermeasures to promote population agglomeration in metropolitan areas. The economic development of a metropolitan area requires the diversification of talent, and accurately determining the concentration of future target groups is crucial. Scientifically planning the future distribution of mobile populations in key areas is essential for shaping an overall population distribution pattern characterized by one core, two belts, and multiple centers. The precise positioning of metropolitan functions, industries, and development within the metropolitan area is essential to stabilize and expand labor force employment. Special attention should be given to the primary mobility needs of different types of labor.

The second aspect involves ensuring the realization of strategies for promoting population agglomeration in the metropolitan area. This is achieved by implementing a development model that increases both the quantity and quality of employment opportunities, thereby increasing the attractiveness of population agglomeration. Additionally, it involves streamlining the channels for population mobility within the metropolitan area to enhance the vitality of population agglomeration. Furthermore, it entails enhancing the capacity to supply high-quality public services and resources within the metropolitan area, ultimately increasing the aggregative power of the population. Strengthening the development of social security capabilities within the metropolitan area is essential to stimulate the intrinsic dynamics of population agglomeration. Moreover, there is a need to intensify research and the optimization of policies for population growth in the metropolitan area, as well as to explore the potential for population agglomeration.
